# Effectiveness and safety of an atropine/midazolam and target controlled infusion propofol-based moderate sedation protocol during percutaneous endoscopic transgastric jejunostomy procedures in Parkinson’s disease: a real-life retrospective observational study

**DOI:** 10.3389/fmed.2023.1233575

**Published:** 2023-09-12

**Authors:** Antonietta Gerarda Gravina, Raffaele Pellegrino, Rosa De Micco, Mirco Dellavalle, Anna Grasso, Giovanna Palladino, Sara Satolli, Massimo Ciaravola, Alessandro Federico, Alessandro Tessitore, Marco Romano, Fausto Ferraro

**Affiliations:** ^1^Department of Precision Medicine, Hepatogastroenterology and Digestive Endoscopy Unit, University of Campania “Luigi Vanvitelli”, Naples, Italy; ^2^Department of Advanced Medical and Surgical Sciences, Neurology Unit, University of Campania “Luigi Vanvitelli”, Naples, Italy; ^3^Department of Woman, Child, General and Specialized Surgery, Anaesthesia and Intensive Care Unit, University of Campania “Luigi Vanvitelli”, Naples, Italy

**Keywords:** endoscopy, percutaneous endoscopic gastrostomy, gastrostomy, PEG-J, Parkinson’s disease, sedation, jejunal tube

## Abstract

Patients with Parkinson’s disease (PD), often elderly with various comorbidities, may require a continuous intestinal infusion of carbidopa/levodopa gel by the placement of a percutaneous endoscopic gastrostomy (PEG) with a jejunal tube (PEG-J) to improve their motor outcome and quality of life. However, it is unclear what is the best procedural sedation protocol for PEG-J procedures. Fifty patients with PD and indication for PEG-J procedure (implantation, replacement, removal) underwent, from 2017 to 2022, a sedation protocol characterized by premedication with atropine (0.01 mg/Kg *i.v.*), midazolam (0.015–0.03 mg/Kg *i.v.*) and induction with bolus propofol (0.5–1 mg/Kg *i.v.*) as well as, finally, sedation with continuous infusion propofol (2–5 mg/Kg/h *i.v.*) by Target Controlled Infusion (TCI) technique. Ninety-eight per cent of patients experienced no intraprocedural or peri-procedural adverse events. All the procedures were technically successful. A good discharge time was recorded. The vital parameters recorded during the procedure did not vary significantly. A PEG-J procedure conducted within 30 min showed a significant advantage over end-tidal carbon dioxide (EtCO_2_). Indeed, the latter showed some predictive behavior (OR: 1.318, 95% CI 1.075–1.615, *p* = 0.008). In the real world, this sedation protocol showed a good safety and effectiveness profile, even with reduced doses of midazolam and a TCI propofol technique in moderate sedation.

## 1. Introduction

Parkinson’s disease (PD) is a neurodegenerative disorder mainly affecting older people; in detail, 3% of people over 65 years old (1). More precisely, it is a progressive disease that results from the death of dopaminergic neurons in the *substantia nigra*. The typical PD symptoms are bradykinesia, muscle rigidity, and tremor at rest. Other possible symptoms are digestion disorder, respiratory dysfunction, circulation problems, and depression. Even though there is no cure for PD, effective treatments can alleviate the symptoms. Oral dopamine replacement therapy (DRT) is the most effective treatment for patients with PD (2). However, DRT is complicated by the evolution of treatment-related motor complications, including wearing-off effects, dyskinesia, and on–off response, which may develop progressively in up to 90% of levodopa-treated patients after 10 years (2). In this disease’s advanced stage, a continuous intestinal infusion of carbidopa/levodopa gel (LCIG) represents an important therapeutic option. The LCIG system provides daily levodopa infusion directly into the proximal jejunum via percutaneous endoscopic gastrostomy (PEG) with a jejunal extension tube (J-tube, PEG-J) connected to a portable infusion pump (3). LCIG delivery into the small intestine overcomes slow and erratic gastric emptying, producing more constant levodopa plasma levels (4). Another PEG J tube placement technique is a peroral route, utilizing only sonographic and fluoroscopic guidance without an endoscope (AbbVie Peg-J) (5). Direct enteral administration of carbidopa/levodopa has decreased plasma concentration variations, resulting in reduced “off” time, improved motor performance, and improved quality of life (4, 6, 7).

Drugs used in anesthesia may interact with PD medication, and there is controversy about the optimal anesthetic management of patients with PD (8). Patients with advanced PD are at risk for exacerbations in the perioperative period. An acute exacerbation is prevented by administering oral levodopa approximately 20 min before inducing anesthesia, which may be repeated intraoperatively and postoperatively every 2 h (9). Fentanyl (10) and propofol (11) have been implicated as causes of movement disorders developing on awakening. Opioid drugs have been reported twice to cause acute stiffening in PD patients, in one case, a dystonic reaction (12) and worsened rigidity and slowness (13).

Most endoscopic procedures are generally performed with the patient under moderate sedation and analgesia, also known as “conscious sedation” (14). Propofol is the primary drug to induce and maintain sedation in upper gastrointestinal tract endoscopy (15, 16).

The purpose of sedation and analgesia is to relieve anxiety, discomfort, or pain and diminish the memory of the event. During therapeutic endoscopy, sedation with propofol and midazolam requires a lower total dose of propofol but otherwise has no superior sedation effectiveness and is associated with a slower post-procedure recovery than sedation with propofol alone (17). A study compared the combination of propofol and fentanyl with midazolam and meperidine in a nonrandomized group of 274 patients undergoing upper endoscopy and colonoscopy. The group receiving propofol and fentanyl had better patient comfort and more profound sedation without an increase in untoward side effects (17). Chan et al. compared a target-controlled infusion of propofol versus intermittent bolus of a sedative cocktail regimen in deep sedation for gastrointestinal endoscopy in 100 patients. TCI with propofol produced less cardiovascular and respiratory suppression than an intermittent bolus of a sedative cocktail regimen in deep sedation for gastrointestinal endoscopy (18). There is no substantial evidence and many studies on the best protocol for PEG-J placement in patients with PD. In addition, prospective and retrospective studies that have reported data on the effectiveness/safety of sedation protocols on PEG-J in patients with PD are almost non-existent.

This retrospective observational study aims to assess a sedation protocol’s real-life effectiveness and safety in patients with PD undergoing PEG-J endoscopic implantation.

## 2. Material and methods

### 2.1. Study setting and pre-operative assessment

This study was set up as retrospective, observational, and real-life. The period considered for the patient’s selection was from 2017 to 2022. Data were retrospectively collected from our clinical PEG-J database. The Units involved were the Neurology Unit, Hepatogastroenterology, and the Department of Anesthesia, Resuscitation, and Intensive Care of the University of Campania Luigi Vanvitelli. Patients with advanced PD and indications for PEG-J placement/replacement to initiate LCIG therapy and those who had been pointed to removing it were included.

Patients underwent a thorough pre-operative anesthesia examination before every PEG-J procedure included in the analysis. The anesthesiologists assessed patients’ operative risk using the American Society of Anesthesiologists Classification (ASA) (19). As part of the same visit, the clinical-demographic variables of patients (gender, age, weight, height, Body Mass Index, i.e., BMI, ASA class, smoking status, comorbidities, presence or absence of cervical hypomobility, presence of edentulous) were collected.

During PEG-J procedures, the time (expressed in seconds) taken by the endoscopist during the performance of esophageal intubation to achieve visualization of the stomach was also recorded. In addition, the time of the complete PEG-J procedure (expressed in minutes) was collected. Another variable was whether the operation performed was the placement/removal of a PEG-J or the replacement of a J-tube on a previously implanted PEG-J. We prescribed, during pre-operative anesthesia examination, laboratory tests (i.e., coagulation tests, routine blood count and biochemistry examination, hepatitis markers) and an electrocardiogram, which were subsequently evaluated. Discontinuation (with any replacement) of some drugs could be prescribed. A platelet counts superior to or equal to 50,000/mm^3^ and an International Normalized Ratio (INR) of 1,4 were generally considered acceptable. The anaesthesiologists took note of the Mallampati score and any other predictor of difficult intubation (20). We excluded pregnant women, patients in whom informed consent could not be obtained, and critically ill patients (ASA IV and V classes).

Our primary outcome was to assess the real-life effectiveness of this sedation protocol (i.e., in terms of procedure success rate) in our study. In addition, our co-primary outcome was to evaluate its safety (i.e., vital signs stability, adverse event rate and the time needed for patient discharge).

### 2.2. Endoscopic technical aspects

PEG-Js were implanted with a *pull* technique (21) performed by two operators (including the first as an endoscopist). In the supine position, the patient was given an EGDS with subsequent complete gastric insufflation with hydrogen dioxide to juxtapose the gastric and abdominal walls by displacing any other viscera that might interpose. The site for the puncture was identified by endoscopic “*transillumination*” and by digital pressure by the second operator. The skin of the selected area was disinfected with iodopovidone and marked with a dermographic pen, and local anesthesia with lidocaine was performed. Utilizing a 21 G-gage needle mounted on a 10 mL syringe filled with 0.9% saline, the puncture was achieved through the selected skin point to inside the gastric lumen with the “*needle aspiration*” technique by which the second operator provided negative pressure employing the syringe and aspirated any air bubbles (also used to check for any interposed viscera that may contraindicate the use of the selected puncture site and indicate the selection of an additional puncture site). Then, the second operator would widen the puncture site with a scalpel to allow the second operator to introduce a trocar (i.e., introducer), while the first operator ensured continuous insufflation of hydrogen dioxide into the gastric lumen.

The introducer was a puncture cannula with a safety (air) valve. Then the needle was pulled out, leaving only the cannula in place. In the latter, a guide wire was introduced inside the stomach that was grasped by an endoscopic loop and carried to the patient’s oral cavity along with the endoscopic loop. That guidewire at its proximal end was tied to the PEG tube, which, lubricated with the internal bumper, was pulled back into the gastric cavity under endoscopic guidance—in this way, pulling from the previously created ostomy so that the internal bumper adhered to the interior cavity. The outer bumper slid over the PEG tube to the outer abdominal wall. The J-tube was then passed through the PEG tube by capturing it internally to the gastric lumen using an endoscopic loop and led endoscopically to the small intestine. Patency was controlled by saline solution infusion. The PEG-J used was the AbbVie™ PEG 15 Fr or 20 Fr and AbbVie™ J 9Fr kits (AbbVie Inc. 1 North Waukegan Road North Chicago, IL 60064 United States Product of Poland).

Absolute contraindications to PEG-J placement considered by the team were lack of apposition between the gastric and abdominal wall (absence of transillumination), severe coagulation disorders (i.e., INR > 5 or platelet count <30,000), abdominal wall infection at the site identified for its placement, ascites greater than grade I, peritonitis or peritoneal carcinosis, previous total gastrectomy, pyloric obstruction, ileum.

### 2.3. PEG-J sedation regimen

The anesthetic technique performed to support PEG-J procedures included both local anesthesia and moderate sedation to provide analgesia and comfort to the patient (with anxiolysis and amnesia) in association with a purposeful response to verbal or tactile stimulation, with the maintenance of spontaneous ventilation and a good cardiovascular function.

The American College of Emergency Physicians (ACEP) defines moderate sedation as “*A technique of administering sedatives or dissociative agents with or without analgesics to induce a state that allows the patient to tolerate unpleasant procedures while maintaining cardiorespiratory function. Procedural sedation and analgesia are intended to result in a depressed level of consciousness that allows the patient to maintain oxygenation and airway control independently*” (22).

All patients were pre-medicated in the recovery room with atropine sulfate monohydrate 0,01 mg/kg *intravenous* and midazolam 0.03–0.05/kg *intravenous*. An intravenous bolus infusion of propofol followed this at a dose of 0.5 to 1 mg/Kg and then a continuous infusion of propofol at a dose of 2.5 mg/Kg per hour. The sedation protocol is summarized in Table 1.

**Table 1:** Proposed percutaneous endoscopic gastrostomy with jejunal extension tube (PEG-J) sedation protocol.

Stage	Drug	Posology
Premedication	Atropine	0.01 mg/Kg *i.v.*
	Midazolam	0.015–0.03 mg/Kg *i.v.*
Induction	Propofol (*bolus*)	0.5–1 mg/Kg *i.v.*
Sedation	Propofol (*Continuous*)	2–5 mg/Kg/h *i.v.*

Heart rate, respiratory rate, pulse oximetry, end-tidal carbon dioxide (EtCO_2_), pulse oximeter oxygen saturation (SpO_2_), and electrocardiogram were monitored continuously in all patients. In addition, blood pressure was recorded every 5 min. Sedation was performed using the Target Controlled Infusion (TCI) protocol, assuring a target concentration of propofol between 2 and 3 gamma/ml. This protocol allowed adequate spontaneous ventilation with oxygen support 3–4 L/min respectively, corresponding to an average of 36 to 40% (FiO_2_) by nasal cannulas, and just in a few cases, assistance with a silicon facial mask was required. We adopted intraprocedural oxygen administration because it was observed to be associated with fewer intraprocedural endoscopic hypoxic episodes in older adults (a large part of our study population) (23). A bite block allowed the introduction of the endoscope. Any emergency devices for airway management (i.e., oropharyngeal airway, laryngeal mask, laryngoscope, endotracheal tube, Frova intubating introducer) and any drug were always disposable. To control the airway, either the mouthpiece or the dual-channel (endoscopic and respiration) LMA® Gastro™ Cuff Pilot™ laryngeal mask was used.

Local anesthesia was performed twice: before the endoscope introduction by 2 to 3 puffs of lidocaine hydrochloride 10% to numb the oropharynx and before the incision and needle insertion in the epigastrium. This latter administration consisted of infiltrating the skin and the superficial fascia of 3 to 4 mL of mepivacaine hydrochloride (20 mg/mL).

**Figure 1:**
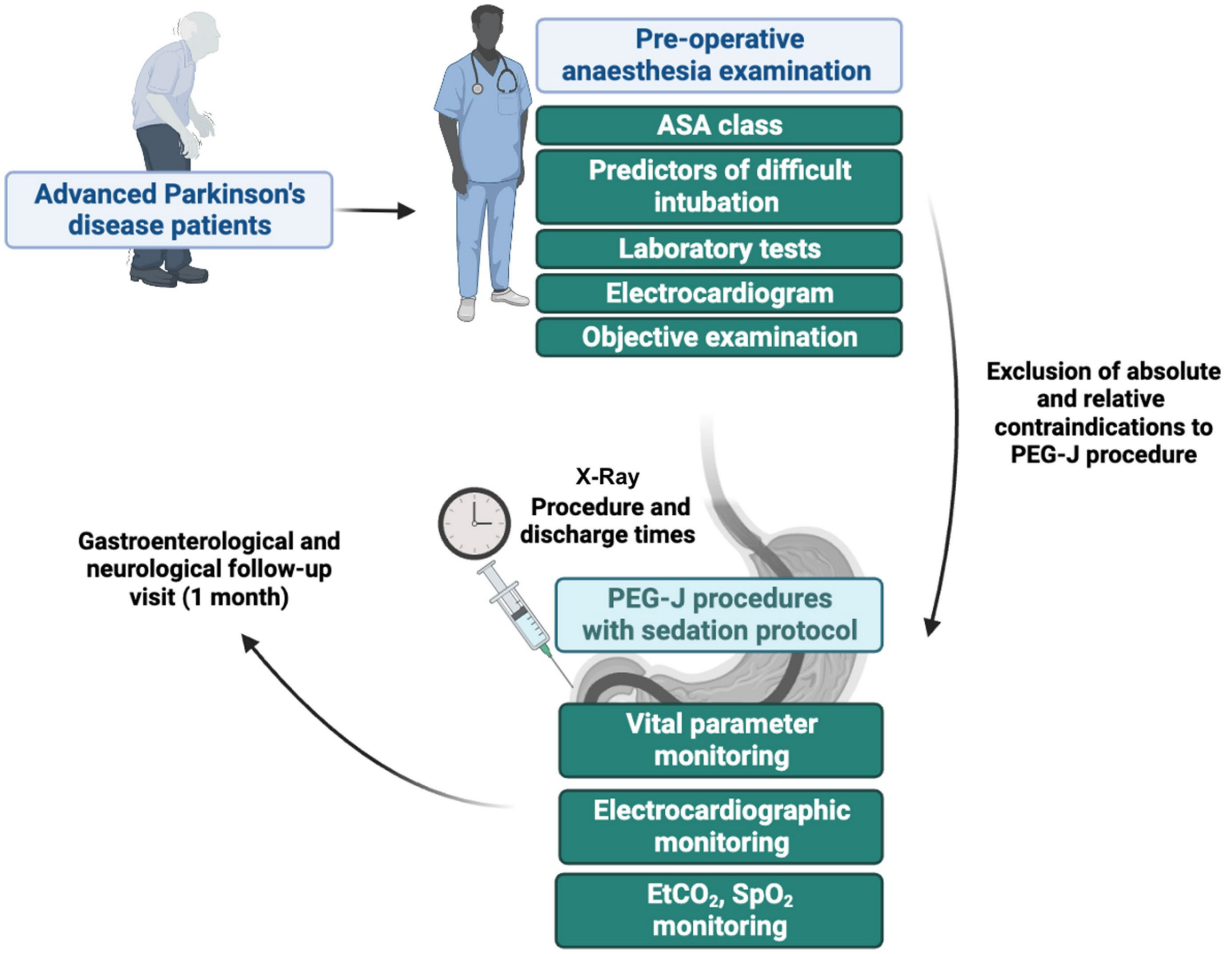
Main steps of the study protocol related to the various steps that patients with Parkinson’s disease had to go through to undergo the percutaneous endoscopic transgastric jejunostomy (PEG-J) procedures. ASA, American Society of Anaesthesiologists; EtCO_2_, end-tidal carbon dioxide; SpO_2_, pulse oximeter oxygen saturation.

The procedures were carried out in a system of care according to which patients were admitted as day hospital patients (at the Hepatogastroenterology or Neurology Unit), underwent a preoperative anesthesiologic examination, and after the PEG-J procedure performed in digestive endoscopy were discharged from endoscopy by the endoscopy and anesthesiology team and referred to their home departments for further monitoring (including X-ray for confirming correct PEG-J positioning) and discharged by the evening of the same day in the absence of complications. However, in the case of the first PEG- J placement, the patient was discharged from the home facility the next day, verifying the absence of PEG-J complications or malfunction. The main elements of the study protocol are summarized in Figure 1.

### 2.4. Statistical analysis

Descriptive statistics were used for the presentation of the data. Continuous variables were expressed as median (interquartile range), and ordinal variables were expressed as numerosity (percentage of total). The normality of the data was evaluated before choosing between parametric and non-parametric tests. The comparisons between subgroups were made by the Mann–Whitney U-test, the Kruskal-Wallis’s test, and the Chi-square test or Fisher’s exact.

A logistic regression model was set to evaluate the predictors of PEG-J procedure duration within 30 min. The latter was assessed according to the goodness of fit according to Hosmer-Lemeshow (as well as according to Cox and Snell R^2^ and Nagelkerke R^2^ values) by expressing the data as an exponential value of B, i.e., exp.(B). The latter was presented as the Odds Ratio (OR), and the risk measure was expressed as the OR and its 95% confidence interval (CI). The value of p accepted as statistically significant was less than 0.05 and if two-tailed (α error = 0.05). Statistical analyzes were conducted using IBM SPSS ® software.

## 3. Results

### 3.1. Population characteristics

**Figure 2:**
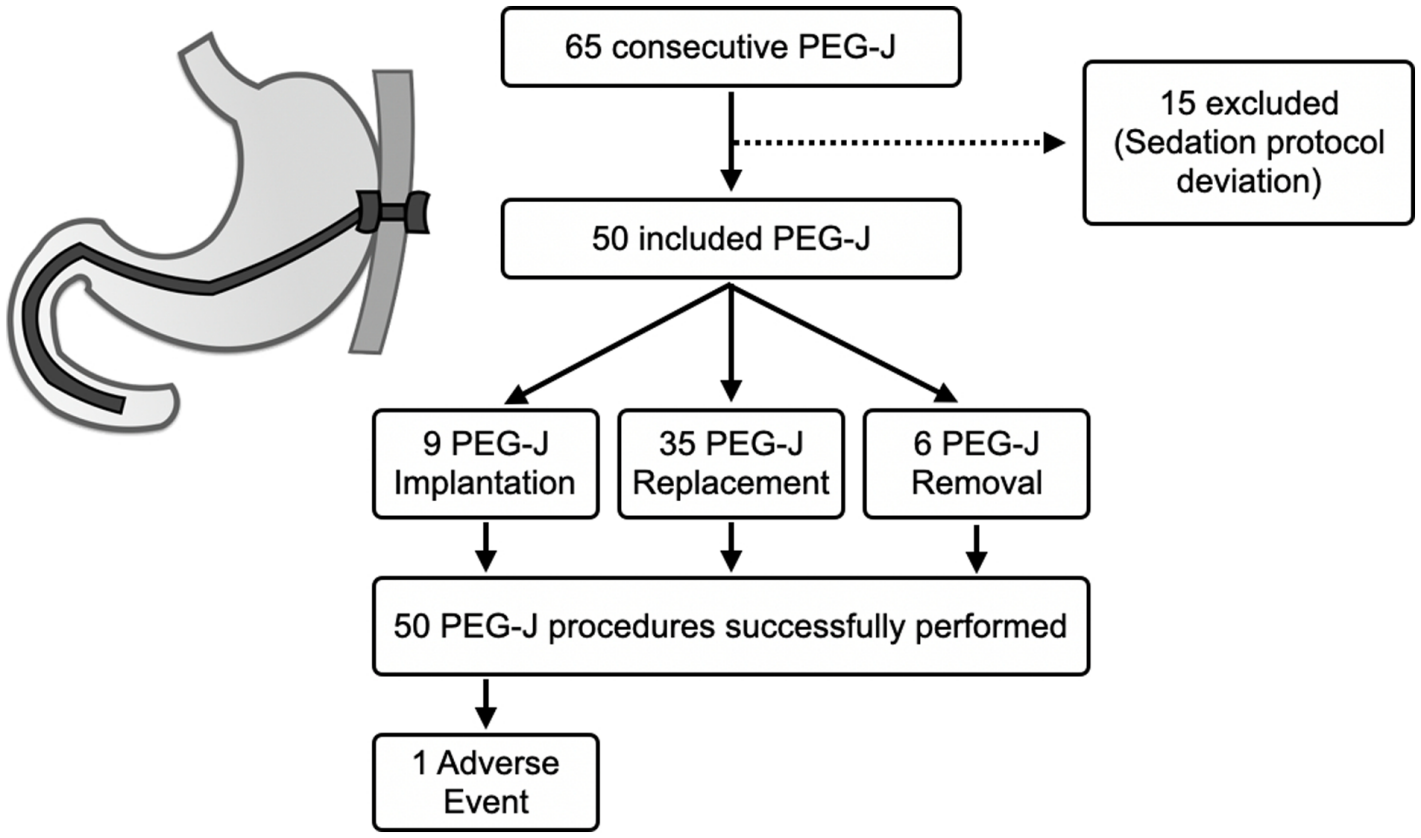
Flow chart related to patient enrolment and reasons for excluding some patients from the study. The data is also filtered by percutaneous endoscopic transgastric jejunostomy (PEG-J) procedure type. Finally, the figure also shows technical success and adverse event rates recorded.

A total of 65 PEG-J procedures in PD patients were performed. In detail, however, 15 patients were not included in the analysis. These patients did not comply with the dosage schedule indicated by our protocol. In the end, 50 patients were included and verified compliance with the protocol. The clinic-demographic characteristics of the patients are summarized in Table 2, while the flowchart of patient inclusion is in Figure 2. The main reasons for patients removing the PEG-J were noncompliance with its use or general complications developed on the pre-existing PEG-J (e.g., bleeding ulcer or buried bumper syndrome). As expected, most of the sample possessed high levels of ASA (i.e., ASA III) because of their median age and associated comorbidities in addition to PD.

**Table 2:** Clinical-demographic characteristics of the sample of patients who underwent percutaneous endoscopic transgastric jejunostomy (PEG-J).

Variable	*n* (%) or median (IQR)
Male	21 (42%)
Female	29 (58%)
Age *(years)*	72 (65.75–75)
Weight *(kilograms)*	64 (55–76)
Height *(centimeters)*	165 (160–173)
Body Mass Index *(kilograms/meters^2^)*	23.74 (20.67–26.68)
Smoker (*yes*)	4 (8%)
Cervical hypomobility (*yes*)	7 (14%)
Edentulous (*yes*)	2 (4%)
Comorbidities	
Arterial hypertension	14 (28%)
Diabetes mellitus	5 (10%)
Chronic obstructive pulmonary disease	3 (6%)
Previous encephalitis	3 (6%)
Previous colorectal carcinoma	2 (4%)
Previous leukemia	1 (2%)
Hysterectomy	2 (4%)
Appendectomy	2 (4%)
Cholecystectomy	1 (2%)
Knee/hip prosthetic implant	2 (4%)
Hypertrophic cardiomyopathy	1 (2%)
I degree atrioventricular block	1 (2%)
Mitral valve stenosis	1 (2%)
Mitral valve insufficiency	1 (2%)
Atrial fibrillation	1 (2%)
Takotsubo syndrome	1 (2%)
Pacemaker implantation for arrhythmia	2 (4%)
Pulmonary fibrosis	1 (2%)
Thyroid nodules	1 (2%)
Cystocele	1 (2%)
Hallux valgus	1 (2%)
Essential thrombocythemia	1 (2%)
Previous ischemic stroke	1 (2%)
Mallampati classification	
Mallampati II	21 (42%)
Mallampati III	28 (56%)
Mallampati IV	1 (2%)
ASA classification	
ASA 2	9 (18%)
ASA 3	41 (82%)
PEG-J rationale	
Positioning	9 (18%)
Replacement	35 (70%)
Removal	6 (12%)

Data are expressed as numerosity (N) and percentage (%) of the total or as median (interquartile range). ASA, American Society of Anesthesiologists.

### 3.2. PEG-J procedures

Concerning the primary outcome, the technical success rate provided by this sedation protocol was 100% (50/50), with no technically failed procedures. On the other hand, regarding safety (i.e., co-primary outcome), we found general stability of clinical parameters and an adverse event rate of 2% (1/50).

The fifty procedures had a median duration of 40 (35–60) minutes and a relatively short post-procedural discharge time of 14 (10–15) minutes. Overall, most procedures (43, 86%) had a sustainable duration because they were conducted within 1 h.

The only adverse event recorded was a laryngeal spasm, resolved without clinical outcomes. Specifically, this was a 66-year-old, diabetic, non-smoking female with a BMI of 26.67 Kg/m^2^ implanting a PEG-J for the first time, with cervical hypomobility, a Mallampati IV and ASA 2, who was given 0.7 mg of atropine, 1 mg of midazolam, 350 mg of propofol for sedation.

Regarding anesthesia monitoring data, the median EtCO_2_ was 24 (32–37.25) mmHg, while that of SpO_2_ was 99 (98–100) %. That is, there was no requirement for 98% of the sample to intervene with devices other than simple nasal cannulas to respond to any onset of hypoxemia.

In follow-up gastroenterological and neurological visits within 1 month following the PEG-J procedure, none of the patients reported additional complications not appreciated in the perioperative period.

In addition, we arbitrarily identified four groups of time ranges of PEG procedure duration (i.e., ≤ 30, ≤ 45, and ≤ 60 min) to determine whether these had an impact on significant anesthesia monitoring variables (i.e., EtCO_2_ and SpO_2_) and on discharge time. Table 3 summarizes our findings.

**Table 3:** EtCO_2_, SpO_2_ values, discharge times and drug doses of patients included in dependence of different Percutaneous Endoscopic transgastric Jejunostomy (PEG-J) procedure duration time ranges.

Time Threshold	PEG-J carried out in the threshold (% of the total)	EtCO_2_ (mmHg)	SpO_2_ (%)	Discharge time (minutes)	Atropine (mg)	Midazolam (mg)	Propofol (mg)
≤ 30 min	11 (22%)	37 (35–40)	100 (98–100)	10 (10–15)	0.7 (0.6–0.8)	1.5 (1–1.5)	200 (170–400)
> 30 min	39 (78%)	33 (30–36)	99 (98–100)	15 (10–15)	0.7 (0.6–0.7)	1 (1–1.5)	250 (180–300)
p-value (95% CI)^a^	-	**0.003 (0.002–0.004)**	0.676 (0.667–0.685)	0.752 (0.743–0.760)	0.782 (0.774–0.790)	0.904 (0.899–0.910)	0.950 (0.946–0.955)
≤ 45 min	31 (62%)	35 (32–37)	99 (98–100)	10 (10–15)	0.7 (0.6–0.7)	1.5 (1–1.5)	200 (170–300)
> 45 min	19 (38%)	32 (30–38)	99 (98–100)	15 (10–15)	0.7 (0.6–0.8)	1 (1–1.5)	250 (200–320)
P-value (95% CI) ^a^	-	0.118 (0.112–0.124)	0.506 (0.497–0.516)	0.052 (0.47–0.56)	0.929 (0.924–0.934)	> 0.9	0.117 (0.111–0.123)
≤ 60 min	43 (86%)	35 (30–37)	99 (98–100)	10 (10–15)	0.7 (0.6–0.8)	1.5 (1–1.5)	200 (170–300)
> 60 min	7 (14%)	33 (32–39)	99 (98–99)	15 (10–15)	0.6 (0.5–0.7)	1 (1–1.5)	300 (250–400)
P-value (95% CI) ^a^	-	0.915 (0.910–0.921)	0.565 (0.555–0.574)	0.140 (0.133–0.147)	0.075 (0.070–0.080)	> 0.9	0.180 (0.172–0.187)

^a^The *p*-value is calculated to assay whether the variable’s value distributes statistically differently, meeting the identified threshold on the two rows above (significant *p*-values are highlighted in bold). Data are expressed as numerosity (N) and percentage (%) of the total or as median (interquartile range).

As observable in the latter, there was a significant advantage on levels (i.e., with higher levels) of EtCO_2_ in conducting the procedure within half an hour (*p* = 0.003). It is also notable how meeting the three-time ranges also equates to having an advantage on post-procedural discharge time and that, for the 45-min range, this advantage is almost significant (*p* = 0.052). SpO_2_ levels remained stable and always acceptable regardless of the duration of the procedure. Moreover, the sedation protocol was not particularly affected by the different time intervals of procedural course (i.e., 30, 45 and 60 min) because the levels of atropine, midazolam and propofol never significantly varied regardless of whether these time intervals were met.

In addition, EtCO_2_ also showed a predictive role of PEG-J procedure duration within 30 min at multivariate analysis (B: 0.276; OR: 1.318, 95% CI 1.075–1.615, *p* = 0.008).

While in addition, stratifying by type of PEG-J procedure (placement, replacement, or removal), the clinical, demographic, and intraprocedural variables do not change significantly (*p* > 0.05, *data not shown*); in contrast, stratifying by gender, there were very different results. In detail, in females, it was found that the procedure duration, 35 (30–35) minutes, was shorter than that of males, 45 (35–57.5) minutes (*p* = 0.037). Weight was also significantly lower in females, 58 (50–68) kg, than in males, 76 (62.5–80) kg (*p* < 0.01). Predictably, as a direct consequence, in females, the doses of midazolam and atropine, 1 (1–1.5) and 0.6 (0.5–0.7) mg/Kg, respectively, were also lower than in males, where they were 1.5 (1.5–1.5) and 0.7 (0.7–0.8) mg/Kg (*p* < 0.01).

Finally, since we reported only in the procedural range within 30 min a significant advantage on one of the parameters of sedation (i.e., EtCO_2_), we assayed exploratively whether clinical-demographic characteristics also differed between those who had or had not complied with this interval. This comparison, however, offered no findings (see Table 4).

**Table 4:** Comparison of clinical and demographic characteristics of patients who did or did not undergo a percutaneous endoscopic gastrostomy with jejunal extension tube (PEG-J) procedure within 30 min.

Variable	≤ 30 min *N* = 11	> 30 min *N* = 39	*P*-value^a^
Male	3 (27.3%)	18 (46.2%)	0.319
Female	8 (72.7%)	21 (53.8%)	
Age *(years)*	73 (71–79)	72 (65–75)	0.158
Weight *(kilograms)*	59 (55–75)	64 (55–78)	0.432
Height *(centimeters)*	160 (160–170)	165 (160–173)	0.237
Body Mass Index *(kilograms/meters^2^)*	22.31 (20.93–29.29)	24.69 (19.59–26.66)	0.842
Smoker (*yes*)	1 (9.1%)	3 (7.7%)	0.999
Cervical hypomobility (*yes*)	2 (18.2%)	5 (12.8%)	0.641
Edentulous (*yes*)	1 (9.1%)	1 (2.6%)	0.395
Mallampati classification			
Mallampati II	6 (54.5%)	15 (38.5%)	0.317
Mallampati III	5 (45.5%)	23 (59%)	
Mallampati IV	-	1 (2.6%)	
ASA classification			
ASA 2	2 (18.2%)	7 (17.9%)	0.986
ASA 3	9 (81.8%)	32 (82.1%)	
PEG-J rationale			
Positioning	1 (9.1%)	8 (20.5%)	0.323
Replacement	1 (9.1%)	5 (12.8%)	
Removal	9 (81.8%)	26 (66.7%)	

^a^The P-value is calculated to assay whether the variable’s value distributes statistically differently, meeting the identified threshold (i.e., 30 min). Data are expressed as numerosity (N) and percentage (%) of the total or as median (interquartile range). ASA: American Society of Anaesthesiologists.

## 4. Discussion

In this real-life observational retrospective study, we showed how a moderate sedation protocol used in clinical practice in our multidisciplinary center was associated with high effectiveness of procedure performance (100%), a good safety profile with the absence of clinically detectable intra- or peri-procedural complications, and within 1 month of follow-up (98%). Lastly, it was associated with a short discharge time of about 10 min. Therefore, we hypothesize that the advantage of discharge time in the case of a PEG-J procedure is potentially attributable to the fact that in case of prolonged timing, there may be an accumulation of the drug and a lengthening of disposal time (especially in patients with PD and slowed metabolism).

In general, endoscopic procedures are associated with a low rate of fatal adverse events, and these tend to occur in older patients with more comorbidities, so the issue of the safety of the procedure, as well as the sedation protocol, is undoubtedly relevant in our setting of patients with advanced PD who are generally older (24). Also, PD patients undergoing PEG may have several comorbidities, as already observed by Arora et al. (25). Indeed, in our sample, there were patients with chronic obstructive pulmonary disease and chronic cough, possible comorbidity to be looked out for in PEG placement (26). Despite this, PEG placement was a relatively safe procedure in experienced hands (27). The estimated PEG-J endoscopic procedure-associated mortality is very low (i.e., 0.5%) (28). Nevertheless, we did not have any untoward effect either intraprocedural or during the follow-up within 1 month from the procedure.

The use of sedation in endoscopic research is getting more widespread to answer to the increasing grade of complexity and duration of endoscopic procedures to offer comfort to the patient regarding analgesia, tolerability, and amnesia. In addition, sedation is also a way to ensure the examination’s quality and safety and improve its outcome (29).

Our results emphasized that the EtCO_2_ parameter was found to be a factor not to be overlooked as a possible determinant of the duration of the endoscopic procedure, confirming, as already outlined in several guidelines, that capnography is a useful supplementary tool in monitoring patients undergoing endoscopic procedures, especially those with comorbidities (15, 16). However, more extensive studies are needed to identify this parameter as a possible predictor of PEG-J procedure completion within 30 min. Although we found such procedural duration prediction behavior within 30 min (i.e., B: 0.276; OR: 1.318, 95% CI 1.075–1.615, *p* = 0.008), it should be pointed out, however, that this analysis conducted in our numerosity requires confirmation in future studies with larger sample size. Another data point to explore is identifying an exact EtCO_2_ threshold to follow during the procedure for this purpose. This assessment also stems from the fact that the numerosity of the groups with procedural durations beyond and within 30 min was different and also took into account the fact that, however, despite this more or less predictive variation in EtCO_2_, we have always kept this capnographic parameter monitored to keep it within normal ranges.

Peveling-Oberhag et al. (30) evaluated capnography monitoring during sedation without an anesthesiologist during PEG placement in a randomized controlled trial, premising that the authors had used a higher propofol dosage than ours (1–1.3 mg/kg bolus followed by 10–30 mg propofol every 2–3 min) and the use of midazolam at the team’s discretion. First, they showed that respiratory complications are not uncommon in PEG. Also, the group of patients with standard monitoring associated with monitored capnography had reduced rates of hypoxemia. Indeed, the sedation protocol in our sample was not related to episodes of hypoxemia (except for the only case of laryngospasm recorded), and this can probably be attributed to a lower dosage of midazolam used and to TCI-guided propofol management. In any case, despite a reduced dosage of midazolam, we did not observe a reduction in the operative effectiveness of the procedure or patient discomfort. However, the previous study did not focus on PD, and the main indication was for oncological reasons.

In another of the few studies available regarding data on sedation in PEG-J (employing the *pull* technique) conducted in 39 patients with amyotrophic lateral sclerosis, the anesthesiologic approach varied widely (31). In 9 cases, the authors employed general anesthesia, while in the rest of the cases, anesthesiologist-controlled sedation (like in our study). However, the drugs used for these procedures differed from ours in that the authors combined propofol with other sedatives/analgesics (i.e., midazolam, remifentanil, ketamine, and piritramide). As a result, propofol dosages were different and lower than ours (i.e., a median of 150 mg of propofol). However, the complication rate in this study was discreetly higher than ours (with one case of pneumoperitoneum and two cases of local infection in addition to five cases of PEG dysfunction and 26 cases of dislocation/dysfunction). However, this comparison must still be interpreted because the indications for PEG-J were different (PD vs. amyotrophic lateral sclerosis).

The shared operative area and the possible impairment of independent respiration make it mandatory for the operator to know how to manage the airway properly to assure the patient’s safety while avoiding intrinsic procedure’s correlated complications: hypoxia, hypercapnia, and aspiration of gastric contents (32). Therefore, to choose the sedation stage, it is necessary to consider patient-dependent, procedure-dependent variables (33) and operator-dependent variables. Generally, quick and easy diagnostic and therapeutic procedures such as PEG-J are easily performed in moderate sedation (34).

More precisely, the result is significant in advanced PD patients to establish an anesthesiologic protocol in the PEG-J procedure for administering LCIG. Intrajejunal infusion of LCIG via PEG-J for long-term treatment of advanced PD patients improves motor fluctuations and quality of life (35–37). Furthermore, performing the procedure under optimal conscious sedation is essential because this procedure’s selected patients are usually not collaborative.

Our anesthesiologic protocol foresees the use of atropine and midazolam in premedication. Atropine helped us to reduce salivation and excessive secretion of the respiratory tract. Midazolam has been used to relieve anxiety, reduce the memory of the event, and reduce propofol dosage (17). Sedation was performed with propofol in TCI, which allowed us to keep patients in spontaneous ventilation with minimum oxygen support by nasal cannulas. In particular regard to analgesia, we preferred to avoid opioid drugs because they have been reported twice to cause acute stiffening in PD patients, in one case a dystonic reaction and, in another, worsened rigidity and slowness (12, 13). Moreover, using opioids associated with propofol increases the possibility of hemodynamic instability and respiratory depression. For this reason, we preferred to have double local analgesia with a 2–3 puff of 10% lidocaine hydrochloride to numb the oropharynx before the introduction of the endoscope and with an epigastric mepivacaine hydrochloride (20 mg/mL) infiltration at the needle introduction point.

In our cases, the sedation protocol relieved or abolished the patient’s discomfort, anxiety, and memory, ensuring compliance with the procedure, patient analgesia, patient safety, and procedure quality. In addition, we observed how a shorter procedural duration was associated in our patient setting with savings in discharge time. The median discharge time was of 30 min. In addition, despite relatively low dosages of midazolam, we had a little anxiolytic effect. In contrast, the patients not only had reasonable procedural anxiolytic control but also, upon awakening, did not manifest psychological or emotional distress.

In our view, it is relevant to collect data on specific sedation protocols considering the increasingly emerging concept of non-anesthesiologist-administered propofol sedation in digestive endoscopy, as it seems to have emerged that propofol gives an effectiveness and safety advantage over midazolam and meperidine for achieving and maintaining a good level of endoscopic sedation (38). A head-to-head comparison of propofol and midazolam showed that propofol was associated with greater endoscopic technical satisfaction despite a higher risk of hypotension (39).

If only considering sedation for routine procedures (i.e., certainly not a PEG-J), recommendations from major guidelines have shown a lack of consensus on the optimal depth of sedation and the optimal sedative agent to use (40). The clinical relevance of this work can be seen in the fact that it adds data on the use of TCI-guided propofol in patients with a higher anesthesia risk than the general population in a background of research on gastrointestinal sedation already in need of evidence overall. In detail, the use of propofol for moderate sedation (i.e., balancing it with benzodiazepines) versus benzodiazepines alone combined with opioids is mainly debated with dissonant recommendations among different international guideline recommendations regarding routine gastrointestinal endoscopy (40–42). In our opinion, sedation during a PEG-J implant procedure is an indispensable element as much for the proper technical success of the process (although relatively safe, it requires several endoscopic and no endoscopic steps) as for the safety and well-being of the patient undergoing it. In addition, however, such a procedure, when framed in the context of patients with neurological comorbidity such as PD, places an even greater need on the anesthesiologists and endoscopists to follow a tailored approach to maintain a good depth of moderate sedation while avoiding the additional risks of general anesthesia (34).

There were several limitations in our study and protocol. First, this is a retrospective, real-life, and not longitudinal series; therefore, a prospective study is necessary to validate our protocol.

Second, conscious sedation would be more accurate if monitored with a bispectral index. Unfortunately, this kind of monitoring was not available in our endoscopy room. Nevertheless, it would help us establish the level of sedation more precisely and improve the procedure’s quality and patient safety. Third, the abolition or compromise of the gag reflex with local pharyngeal anesthesia, intravenous sedation and splinting of the top of the esophageal sphincter from the endoscope all predispose to aspiration of gastric contents. Finally, pulmonary aspiration is a life-threatening complication of upper gastrointestinal endoscopy. Patients with advanced PD have an increased risk of developing this complication. For these reasons, it would be more appropriate to use specific medical devices to protect the trachea or lungs from the threat of aspiration. Our study, however, addressed only the *pull* technique for PEG placement. However, the push technique can also be contemplated, especially in PEG-J replacements (i.e., PEG-J insertion through the ostomy of a previous PEG-J). For example, in a retrospective study, Simoni et al. (43) collected data from 156 PEG-J procedures, most of which had been performed with push, a technique that does not require special sedation procedures. The authors’ data showed a reduction in the rate of ostomy complications in patients with replacement by push technique. Therefore, the replacement setting in centers that have experienced the push technique should, in any case, be looked at with great interest.

In conclusion, the sedation protocol described in this report for PD patients undergoing endoscopic PEG-J positioning has shown good real-life effectiveness, safety, and timesaving and might be considered in this clinical setting.

## Data availability statement

The original contributions presented in the study are included in the article/supplementary material, further inquiries can be directed to the corresponding author.

## Ethics statement

Ethical approval was not required for the studies involving humans because the study is retrospective and there is deidentified data. In addition, the study was conducted in accordance with the Declaration of Helsinki. The studies were conducted in accordance with the local legislation and institutional requirements. The participants provided their written informed consent to participate in this study.

## Author contributions

AGG, RP, AF, AT, MR, and FF: study concept and design. AGG, RP, RD, MD, AG, SS, MC, AF, AT, MR, and FF: acquisition of data and interpretation of data. AGG and RP: formal analysis. AGG, RP, and FF: drafting of the manuscript. All authors contributed to the article and approved the submitted version.

## Conflict of interest

The authors declare that the research was conducted in the absence of any commercial or financial relationships that could be construed as a potential conflict of interest.

## Publisher’s note

All claims expressed in this article are solely those of the authors and do not necessarily represent those of their affiliated organizations, or those of the publisher, the editors and the reviewers. Any product that may be evaluated in this article, or claim that may be made by its manufacturer, is not guaranteed or endorsed by the publisher.
